# Dimethyl­ammonium 4-nitro­phenolate–4-nitro­phenol (1/1)

**DOI:** 10.1107/S1600536810008317

**Published:** 2010-03-10

**Authors:** Jing-Mei Xiao

**Affiliations:** a Ordered Matter Science Research Center, College of Chemistry and Chemical Engineering, Southeast University, Nanjing 211189, People’s Republic of China

## Abstract

The title compound, C_2_H_8_N^+^·C_6_H_4_NO_3_^−^·C_6_H_5_NO_3_, was synthesized from dimethyl­amine and 4-nitro­phenol in an overall yield of 85%. The dihdral angles between the nphenyl rings and their attached nitro groups are 5.7 (6) and 2.5 (7)°. In the crystal, there are strong hydrogen bonds between the ammonium group and the nitro­phenol and nitro­phenolate O atoms, and between the nitro­phenol and nitro­phenolate O atoms, forming a chain along the *b*-axis direction.

## Related literature

For background to dialectric behaviour, see: Horiuchi *et al.* (2007 ▶); Kumai *et al.* (2006 ▶).
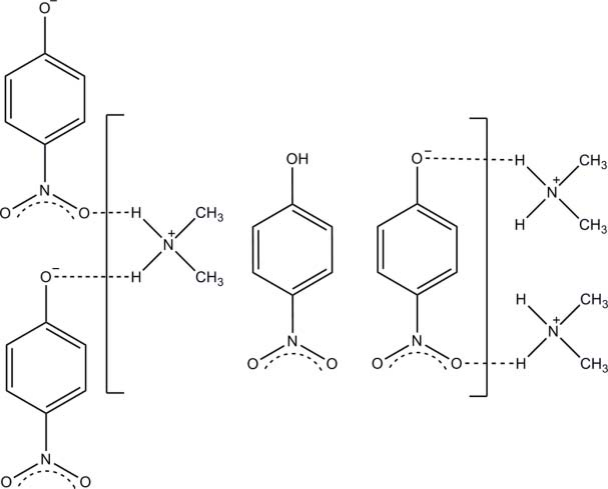


## Experimental

### 

#### Crystal data

C_2_H_8_N^+^·C_6_H_4_NO_3_^−^·C_6_H_5_NO_3_
*M* *_r_* = 323.31
Monoclinic, 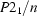

*a* = 6.3185 (10) Å
*b* = 16.8867 (10) Å
*c* = 15.1015 (14) Å
β = 101.928 (10)°
*V* = 1576.5 (3) Å^3^
*Z* = 4
Mo *K*α radiation
μ = 0.11 mm^−1^
*T* = 293 K
0.40 × 0.30 × 0.20 mm

#### Data collection

Rigaku Mercury2 diffractometer
Absorption correction: multi-scan (*CrystalClear*; Rigaku, 2005 ▶) *T*_min_ = 0.960, *T*_max_ = 0.977
15857 measured reflections
3617 independent reflections
1474 reflections with *I* > 2σ(*I*)
*R*_int_ = 0.134

#### Refinement

*R*[*F* ^2^ > 2σ(*F* ^2^)] = 0.076
*wR*(*F* ^2^) = 0.212
*S* = 1.00
3617 reflections
214 parameters
H atoms treated by a mixture of independent and constrained refinement
Δρ_max_ = 0.19 e Å^−3^
Δρ_min_ = −0.19 e Å^−3^

### 

Data collection: *CrystalClear* (Rigaku, 2005 ▶); cell refinement: *CrystalClear*; data reduction: *CrystalClear*; program(s) used to solve structure: *SHELXS97* (Sheldrick, 2008 ▶); program(s) used to refine structure: *SHELXL97* (Sheldrick, 2008 ▶); molecular graphics: *SHELXTL* (Sheldrick, 2008 ▶); software used to prepare material for publication: *SHELXL97*.

## Supplementary Material

Crystal structure: contains datablocks I, global. DOI: 10.1107/S1600536810008317/ez2184sup1.cif

Structure factors: contains datablocks I. DOI: 10.1107/S1600536810008317/ez2184Isup2.hkl

Additional supplementary materials:  crystallographic information; 3D view; checkCIF report

## Figures and Tables

**Table 1:** Hydrogen-bond geometry (Å, °)

*D*—H⋯*A*	*D*—H	H⋯*A*	*D*⋯*A*	*D*—H⋯*A*
N3—H3*B*⋯O4^i^	0.90	2.11	3.000 (4)	170
N3—H3*C*⋯O1	0.90	1.82	2.704 (4)	165
O2—H1⋯O1^ii^	0.91 (4)	1.64 (4)	2.548 (3)	175 (4)

Symmetry codes: (i) 
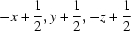
; (ii) 
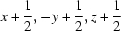
.

**Fig. 1.**
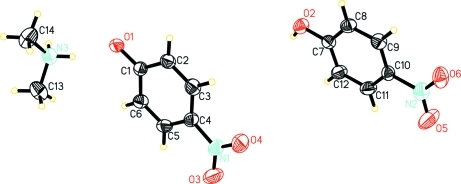
The molecular structure of the title compound, showing the atomic numbering scheme. Displacement ellipsoids are drawn at the 30% probability level.

**Fig. 2.**
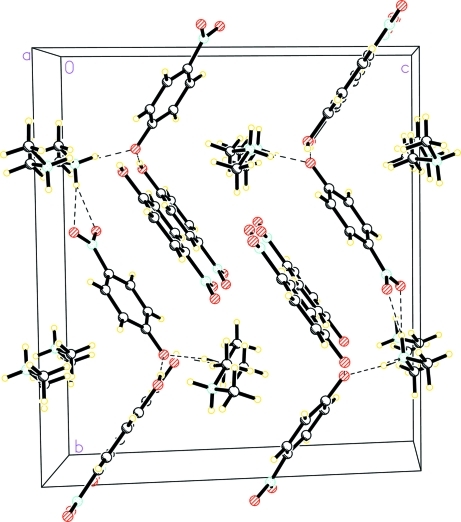
A view of the packing of the title compound, stacking along the a axis. Dashed lines indicate hydrogen bonds.

## supplementary crystallographic information

### Comment

Studying dielectric behavior is the basic method of characterization for potential ferroelectrics in which there is a dielectric anomaly at the transition temperature (Kumai *et al.*, 2006; Horiuchi *et al.* 2007). In our case, unfortunately, the title compound has no dielectric disuniformity between 80 K to 350 K (m.p. 381–365 K), however its structure is reported here.

In this report we have established unambiguously the structure of dimethylammonium 4-nitrophenolate-4-nitrophenol (1/1) in the solid state by X-ray diffraction analysis, as shown in Fig. 1. Intermolecular N—H···O, N—H···N and O—H···O hydrogen bonds are found between the dimethylammonium cation and 4-nitrophenolate anion and the 4-nitrophenolate cation and 4-nitrophenol molecule, forming a chain along the b-axis.

### Experimental

The title complex was obtained by mixing dimethylamine water solution (33%, 0.68 g) and 4-nitrophenol (5 mmol, 0.695 g) in 15 ml ethanol, in the stoichiometric ratio 1:1. After a few weeks, yellow crystals were obtained by slow evaporation.

### Refinement

All H-atoms were located from difference maps and those on N and C were positioned geometrically and refined using a riding model with (N—H = 0.90, C—H = 0.93 and 0.96 Å for aromatic, methyl H respectively) and constrained to ride on their parent atoms with *U*_iso_(H) = xU_eq_(C), where *x* = 1.5 for methyl H and *x* = 1.2 for all other H atoms. Atom H1 on atom O2 was refined isotropically.

### Crystal data

**Table:**

C_2_H_8_N^+^·C_6_H_4_NO_3_^−^·C_6_H_5_NO_3_	*F*(000) = 680
*M**_r_* = 323.31	*D*_x_ = 1.362 Mg m^−^^3^
Monoclinic, *P*2_1_/*n*	Mo *K*α radiation, λ = 0.71073 Å
Hall symbol: -P 2yn	Cell parameters from 0 reflections
*a* = 6.3185 (10) Å	θ = 2.7–27.3°
*b* = 16.8867 (10) Å	µ = 0.11 mm^−^^1^
*c* = 15.1015 (14) Å	*T* = 293 K
β = 101.928 (10)°	Prism, yellow
*V* = 1576.5 (3) Å^3^	0.40 × 0.30 × 0.20 mm
*Z* = 4	

### Data collection

**Table:**

Rigaku Mercury2 diffractometer	3617 independent reflections
Radiation source: fine-focus sealed tube	1474 reflections with *I* > 2σ(*I*)
graphite	*R*_int_ = 0.134
Detector resolution: 13.6612 pixels mm^-1^	θ_max_ = 27.5°, θ_min_ = 3.0°
CCD_Profile_fitting scans	*h* = −8→8
Absorption correction: multi-scan (*CrystalClear*; Rigaku, 2005)	*k* = −21→21
*T*_min_ = 0.960, *T*_max_ = 0.977	*l* = −19→19
15857 measured reflections	

### Refinement

**Table:**

Refinement on *F*^2^	Primary atom site location: structure-invariant direct methods
Least-squares matrix: full	Secondary atom site location: difference Fourier map
*R*[*F*^2^ > 2σ(*F*^2^)] = 0.076	Hydrogen site location: inferred from neighbouring sites
*wR*(*F*^2^) = 0.212	H atoms treated by a mixture of independent and constrained refinement
*S* = 1.00	*w* = 1/[σ^2^(*F*_o_^2^) + (0.0781*P*)^2^] where *P* = (*F*_o_^2^ + 2*F*_c_^2^)/3
3617 reflections	(Δ/σ)_max_ < 0.001
214 parameters	Δρ_max_ = 0.19 e Å^−^^3^
0 restraints	Δρ_min_ = −0.18 e Å^−^^3^

### Special details

**Table:**

Geometry. All e.s.d.'s (except the e.s.d. in the dihedral angle between two l.s. planes) are estimated using the full covariance matrix. The cell e.s.d.'s are taken into account individually in the estimation of e.s.d.'s in distances, angles and torsion angles; correlations between e.s.d.'s in cell parameters are only used when they are defined by crystal symmetry. An approximate (isotropic) treatment of cell e.s.d.'s is used for estimating e.s.d.'s involving l.s. planes.
Refinement. Refinement of *F*^2^ against ALL reflections. The weighted *R*-factor *wR* and goodness of fit *S* are based on *F*^2^, conventional *R*-factors *R* are based on *F*, with *F* set to zero for negative *F*^2^. The threshold expression of *F*^2^ > σ(*F*^2^) is used only for calculating *R*-factors(gt) *etc*. and is not relevant to the choice of reflections for refinement. *R*-factors based on *F*^2^ are statistically about twice as large as those based on *F*, and *R*- factors based on ALL data will be even larger.

### Fractional atomic coordinates and isotropic or equivalent isotropic displacement parameters (Å^2^)

**Table:**

	*x*	*y*	*z*	*U*_iso_*/*U*_eq_	
C1	0.1166 (5)	0.16858 (18)	0.2568 (2)	0.0476 (8)	
C2	−0.0163 (5)	0.13397 (19)	0.3106 (2)	0.0551 (9)	
H2	−0.1503	0.1564	0.3115	0.066*	
C3	0.0485 (6)	0.0682 (2)	0.3612 (2)	0.0622 (10)	
H3A	−0.0433	0.0451	0.3946	0.075*	
C4	0.2496 (6)	0.03589 (19)	0.3631 (2)	0.0541 (9)	
C5	0.3856 (6)	0.06954 (19)	0.3122 (2)	0.0566 (9)	
H5	0.5208	0.0474	0.3131	0.068*	
C6	0.3216 (5)	0.13475 (19)	0.2612 (2)	0.0530 (9)	
H6	0.4154	0.1575	0.2284	0.064*	
C7	0.1727 (5)	0.15350 (19)	0.7572 (2)	0.0492 (9)	
C8	−0.0224 (5)	0.1312 (2)	0.7775 (2)	0.0563 (9)	
H8	−0.1464	0.1606	0.7553	0.068*	
C9	−0.0337 (6)	0.0659 (2)	0.8302 (2)	0.0575 (9)	
H9	−0.1655	0.0506	0.8431	0.069*	
C10	0.1485 (6)	0.02359 (19)	0.8637 (2)	0.0499 (9)	
C11	0.3448 (6)	0.0449 (2)	0.8463 (3)	0.0668 (11)	
H11	0.4683	0.0156	0.8696	0.080*	
C12	0.3560 (6)	0.1109 (2)	0.7933 (3)	0.0642 (11)	
H12	0.4888	0.1267	0.7819	0.077*	
C13	−0.0771 (7)	0.2605 (2)	−0.0167 (3)	0.0913 (14)	
H13A	−0.0713	0.2802	−0.0758	0.137*	
H13B	−0.1768	0.2918	0.0085	0.137*	
H13C	−0.1244	0.2064	−0.0214	0.137*	
C14	0.3086 (7)	0.2208 (2)	0.0111 (3)	0.0861 (13)	
H14A	0.2750	0.1653	0.0100	0.129*	
H14B	0.4451	0.2299	0.0514	0.129*	
H14C	0.3168	0.2379	−0.0488	0.129*	
N1	0.3125 (7)	−0.03649 (18)	0.4114 (2)	0.0694 (9)	
N2	0.1366 (7)	−0.04734 (19)	0.9189 (2)	0.0692 (9)	
N3	0.1396 (5)	0.26538 (15)	0.0422 (2)	0.0671 (9)	
H3B	0.1793	0.3166	0.0478	0.081*	
H3C	0.1310	0.2477	0.0976	0.081*	
O1	0.0540 (4)	0.23037 (12)	0.20599 (14)	0.0553 (7)	
O2	0.1749 (4)	0.21762 (15)	0.70479 (17)	0.0650 (8)	
O3	0.4907 (5)	−0.06544 (16)	0.4107 (2)	0.0896 (10)	
O4	0.1846 (5)	−0.06924 (15)	0.45180 (19)	0.0896 (10)	
O5	0.3015 (6)	−0.08400 (18)	0.9483 (2)	0.1126 (12)	
O6	−0.0419 (5)	−0.06671 (15)	0.93146 (17)	0.0791 (9)	
H1	0.313 (7)	0.233 (2)	0.704 (3)	0.104 (16)*	

### Atomic displacement parameters (Å^2^)

**Table:**

	*U*^11^	*U*^22^	*U*^33^	*U*^12^	*U*^13^	*U*^23^
C1	0.057 (2)	0.0400 (19)	0.045 (2)	−0.0029 (17)	0.0077 (17)	−0.0043 (16)
C2	0.061 (2)	0.048 (2)	0.060 (2)	0.0018 (18)	0.0225 (19)	−0.0010 (18)
C3	0.074 (3)	0.051 (2)	0.067 (3)	−0.007 (2)	0.028 (2)	0.0028 (19)
C4	0.069 (2)	0.0402 (19)	0.053 (2)	0.0022 (18)	0.011 (2)	0.0003 (17)
C5	0.060 (2)	0.046 (2)	0.064 (2)	0.0070 (18)	0.012 (2)	−0.0058 (19)
C6	0.053 (2)	0.045 (2)	0.063 (2)	−0.0023 (17)	0.0166 (19)	0.0012 (18)
C7	0.052 (2)	0.0426 (19)	0.055 (2)	−0.0042 (17)	0.0154 (18)	−0.0032 (17)
C8	0.046 (2)	0.053 (2)	0.067 (2)	0.0017 (17)	0.0077 (19)	0.0025 (19)
C9	0.051 (2)	0.058 (2)	0.065 (2)	−0.0081 (19)	0.014 (2)	−0.002 (2)
C10	0.057 (2)	0.046 (2)	0.048 (2)	−0.0057 (18)	0.0156 (18)	0.0008 (16)
C11	0.055 (2)	0.061 (2)	0.087 (3)	0.0125 (19)	0.020 (2)	0.011 (2)
C12	0.054 (2)	0.057 (2)	0.086 (3)	0.0044 (19)	0.024 (2)	0.014 (2)
C13	0.099 (4)	0.077 (3)	0.088 (3)	−0.004 (2)	−0.004 (3)	0.014 (2)
C14	0.098 (3)	0.087 (3)	0.083 (3)	−0.012 (3)	0.041 (3)	−0.013 (2)
N1	0.102 (3)	0.046 (2)	0.059 (2)	0.003 (2)	0.013 (2)	0.0026 (16)
N2	0.089 (3)	0.055 (2)	0.065 (2)	−0.014 (2)	0.018 (2)	−0.0048 (17)
N3	0.102 (3)	0.0431 (17)	0.0586 (19)	−0.0083 (17)	0.022 (2)	0.0005 (15)
O1	0.0664 (16)	0.0450 (13)	0.0580 (15)	0.0080 (12)	0.0205 (12)	0.0035 (12)
O2	0.0596 (18)	0.0565 (16)	0.0798 (18)	−0.0041 (13)	0.0167 (15)	0.0149 (13)
O3	0.095 (2)	0.0640 (18)	0.110 (2)	0.0230 (17)	0.022 (2)	0.0174 (16)
O4	0.132 (3)	0.0583 (17)	0.087 (2)	0.0032 (17)	0.044 (2)	0.0174 (15)
O5	0.102 (3)	0.084 (2)	0.148 (3)	0.0146 (19)	0.018 (2)	0.054 (2)
O6	0.098 (2)	0.0679 (18)	0.078 (2)	−0.0276 (16)	0.0314 (17)	0.0043 (14)

### Geometric parameters (Å, °)

**Table:**

C1—O1	1.307 (3)	C10—N2	1.471 (4)
C1—C6	1.405 (4)	C11—C12	1.382 (4)
C1—C2	1.410 (4)	C11—H11	0.9300
C2—C3	1.362 (4)	C12—H12	0.9300
C2—H2	0.9300	C13—N3	1.473 (5)
C3—C4	1.378 (5)	C13—H13A	0.9600
C3—H3A	0.9300	C13—H13B	0.9600
C4—C5	1.387 (4)	C13—H13C	0.9600
C4—N1	1.436 (4)	C14—N3	1.462 (4)
C5—C6	1.357 (4)	C14—H14A	0.9600
C5—H5	0.9300	C14—H14B	0.9600
C6—H6	0.9300	C14—H14C	0.9600
C7—O2	1.343 (4)	N1—O3	1.229 (4)
C7—C12	1.376 (4)	N1—O4	1.239 (4)
C7—C8	1.382 (4)	N2—O5	1.214 (4)
C8—C9	1.371 (4)	N2—O6	1.226 (4)
C8—H8	0.9300	N3—H3B	0.9000
C9—C10	1.360 (4)	N3—H3C	0.9000
C9—H9	0.9300	O2—H1	0.91 (4)
C10—C11	1.368 (4)		
			
O1—C1—C6	121.1 (3)	C10—C11—H11	120.6
O1—C1—C2	121.7 (3)	C12—C11—H11	120.6
C6—C1—C2	117.2 (3)	C7—C12—C11	120.7 (3)
C3—C2—C1	121.0 (3)	C7—C12—H12	119.7
C3—C2—H2	119.5	C11—C12—H12	119.7
C1—C2—H2	119.5	N3—C13—H13A	109.5
C2—C3—C4	120.2 (3)	N3—C13—H13B	109.5
C2—C3—H3A	119.9	H13A—C13—H13B	109.5
C4—C3—H3A	119.9	N3—C13—H13C	109.5
C3—C4—C5	120.0 (3)	H13A—C13—H13C	109.5
C3—C4—N1	120.3 (3)	H13B—C13—H13C	109.5
C5—C4—N1	119.4 (3)	N3—C14—H14A	109.5
C6—C5—C4	120.1 (3)	N3—C14—H14B	109.5
C6—C5—H5	120.0	H14A—C14—H14B	109.5
C4—C5—H5	120.0	N3—C14—H14C	109.5
C5—C6—C1	121.3 (3)	H14A—C14—H14C	109.5
C5—C6—H6	119.3	H14B—C14—H14C	109.5
C1—C6—H6	119.3	O3—N1—O4	121.2 (3)
O2—C7—C12	122.9 (3)	O3—N1—C4	119.5 (4)
O2—C7—C8	117.9 (3)	O4—N1—C4	119.3 (4)
C12—C7—C8	119.1 (3)	O5—N2—O6	123.7 (3)
C9—C8—C7	120.2 (3)	O5—N2—C10	118.8 (4)
C9—C8—H8	119.9	O6—N2—C10	117.5 (4)
C7—C8—H8	119.9	C14—N3—C13	115.2 (3)
C10—C9—C8	119.8 (3)	C14—N3—H3B	108.5
C10—C9—H9	120.1	C13—N3—H3B	108.5
C8—C9—H9	120.1	C14—N3—H3C	108.5
C9—C10—C11	121.4 (3)	C13—N3—H3C	108.5
C9—C10—N2	120.0 (3)	H3B—N3—H3C	107.5
C11—C10—N2	118.6 (3)	C7—O2—H1	111 (3)
C10—C11—C12	118.7 (3)		
			
O1—C1—C2—C3	178.1 (3)	C8—C9—C10—N2	−178.9 (3)
C6—C1—C2—C3	−3.0 (5)	C9—C10—C11—C12	−0.2 (5)
C1—C2—C3—C4	2.3 (5)	N2—C10—C11—C12	179.1 (3)
C2—C3—C4—C5	−1.0 (5)	O2—C7—C12—C11	−179.5 (3)
C2—C3—C4—N1	−175.7 (3)	C8—C7—C12—C11	2.4 (5)
C3—C4—C5—C6	0.6 (5)	C10—C11—C12—C7	−1.2 (5)
N1—C4—C5—C6	175.3 (3)	C3—C4—N1—O3	178.4 (3)
C4—C5—C6—C1	−1.4 (5)	C5—C4—N1—O3	3.7 (5)
O1—C1—C6—C5	−178.5 (3)	C3—C4—N1—O4	−0.5 (5)
C2—C1—C6—C5	2.6 (5)	C5—C4—N1—O4	−175.2 (3)
O2—C7—C8—C9	179.5 (3)	C9—C10—N2—O5	−179.8 (4)
C12—C7—C8—C9	−2.3 (5)	C11—C10—N2—O5	1.0 (5)
C7—C8—C9—C10	0.9 (5)	C9—C10—N2—O6	1.4 (5)
C8—C9—C10—C11	0.3 (5)	C11—C10—N2—O6	−177.9 (3)

### Hydrogen-bond geometry (Å, °)

**Table:**

*D*—H···*A*	*D*—H	H···*A*	*D*···*A*	*D*—H···*A*
N3—H3B···O4^i^	0.90	2.11	3.000 (4)	170
N3—H3C···O1	0.90	1.82	2.704 (4)	165
O2—H1···O1^ii^	0.91 (4)	1.64 (4)	2.548 (3)	175 (4)

Symmetry codes: (i) −*x*+1/2, *y*+1/2, −*z*+1/2; (ii) *x*+1/2, −*y*+1/2, *z*+1/2.
